# Influence of extruded injector nozzle on fuel mixing and mass diffusion of multi fuel jets in the supersonic cross flow: computational study

**DOI:** 10.1038/s41598-023-39306-z

**Published:** 2023-07-26

**Authors:** Seyyed Amirreza Abdollahi, Ghazal Rajabikhorasani, As’ad Alizadeh

**Affiliations:** 1 Faculty of Mechanical Engineering, University of Tabriz, Tabriz, Iran; 2 Department of Mechanical and Aerospace Engineering, College of Engineering and Applied Sciences, Western Michigan University, Kalamazoo, MI 49008 USA; 3 Department of Civil Engineering, College of Engineering, Cihan University-Erbil, Erbil, Iraq

**Keywords:** Energy science and technology, Engineering

## Abstract

The efficient injection system has a great role on the overall enactment of air breathing propulsion systems at supersonic flow. In this work, the usage of extruded multi-injectors in the fuel distribution and mixing through the combustor is fully investigated. The usage of the extruded nozzles considerably intensifies the formation of the vortices nearby the injectors and this research has tried to visualize the role of these vortices on the diffusion of the fuel jet through the combustor of the scramjet. The influences of the jet space on the strength of produced circulations are fully discussed. The simulation of the high-speed air stream moving the combustion chamber with extruded nozzles is done via Computational Fluid dynamics. Based on our computational data, the use of extruded multi-jets enhances the penetration and diffusion of the hydrogen cross jet in supersonic airflow. Increasing the gap between injectors improves fuel mixing performance by up to 27% downstream of the jets, primarily by enhancing the lateral penetration of the fuel jet.

## Introduction

The most significant advance in propulsion technology for accessing space is the invention of scramjet engines^1–5^. A launch vehicle with a scramjet engine could work over part of the launcher's intra-atmospheric flight with more sophisticated effectiveness than a similar rocket via releasing mass for increased payload or reusability^5–7^. To show the high impacts of this device in real applications, a scramjet could operate in hypervelocity speeds up to Mach 12. Due to these advantages of scramjet engines, this technique has been investigated considerably in recent decades. In fact, this is the only known technique for accessing outer space. Besides, this propulsion system produced the required power and thrust for high-speed flight^8–10^.

Scramjet engine includes four main stages: Inlet (compression), diffuser; burner and exhaust nozzle. In the first stage, the pressure of the supersonic air is amplified and then, the fuel mixing happens in the diffuser and the auto-ignition process happens in the burner and high enthalpy gas is exhausted with high momentum from the outlet nozzle^11–13^. The main difference between this engine with ramjet engine is the velocity of the incoming air inside the chamber of combustion where the fuel injection and auto-ignition process happen^14–16^. In the ramjet engine, the velocity of the airflow reduces to subsonic speed although its initial speed could be up to Mach = 2. In fact, the mixing and ignition happen in the subsonic domain^17^. However, the supersonic flow preserved its velocity in the combustor of the scramjet engine and fuel injection and mixing progression in this section happens in the supersonic velocity. This different condition makes these processes more complex and complicated by reason of the higher velocity of the incoming air^18–20^. In fact, the high velocity of air limited the time of fuel mixing while several shocks happened because of air jet interactions with jet plume. To resolve these difficulties, few practical systems have been established to preserve the efficiency of this type of engine even in higher velocities^21–23^.

As mentioned, the distribution and combustion of fuel is done in the combustion chamber and the mechanism of the fuel injection and mixing happens via the injection methodology (active or passive)^24,25^. In active concept, vibration splitter, pulse jet and wavy wall are the conventional procedure for the efficient injection of fuel^26–28^. In these methods, forced excitation of a large-scale mechanism is used. On the other hand, ramps, lobe mixers, taps, cavities and vanes are used for the shot of the fuel inside the combustion chamber. Besides, the transverse and counter flow jet are the two most popular techniques in this category^29–31^.

The application of the transverse jets is extensively investigated in the context due to considerable benefits in mixing the fuel^32,33^. Replacement of a single jet with multiple equivalent jets also enhances the performance of this approach for fuel diffusion by the usage of the vortex within gaps of injectors^34,35^. Owing to these advantages, this work has tried to develop this methodology via applying extruded nozzles of multi-jets in combustion chambers^32,36^. In the proposed configurations, fuel nozzles are positioned from a higher level that may improve the fuel mixing and augment the vortex feature in the gap of injectors.

In the present research study, the computational fluid dynamic is selected for the investigation of the flow around the extruded multiple jets at the supersonic free stream. Flow analyses are presented to disclose the impacts of vortices on the mechanism of fuel distribution encounter supersonic cross-air stream. The effects of jet spaces on jet diffusion and mixing are entirely investigated in this research. A comparison of the fuel mixing index and circulation strength is also done for achieving the optimum condition for hydrogen injection.

## Governing equations and numerical technique

The computational visualization of the high-speed flow is mainly done by means of resolving RANS equations with SST turbulence model^37,38^. The simulation of the hydrogen jet in the air stream is done by coupling the species transport equation with the main governing equations^39^. Besides, the compressible effects and jet interactions result in the shock waves and this is achieved by the coupling of the energy equation with primary equations. The ideal gas assumption is a reasonable choice for the calculation of the density in our problem. The main governing equations of our problem were fully presented in the prior published works^40,41^. A fully implicit approach is used for solving our main governing equations^42^.

**Figure 1:**
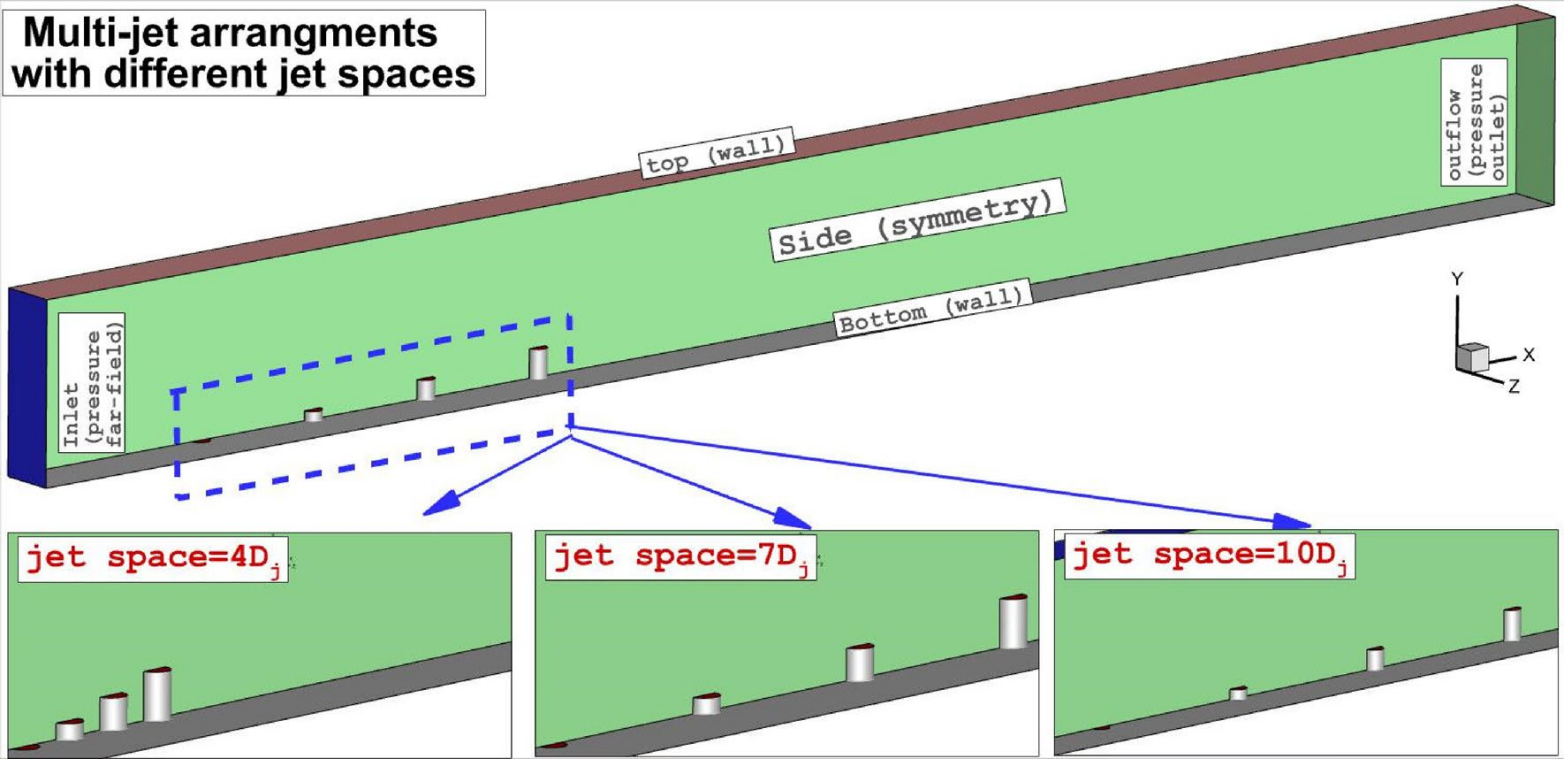
Boundary conditions and model description.

**Figure 2:**
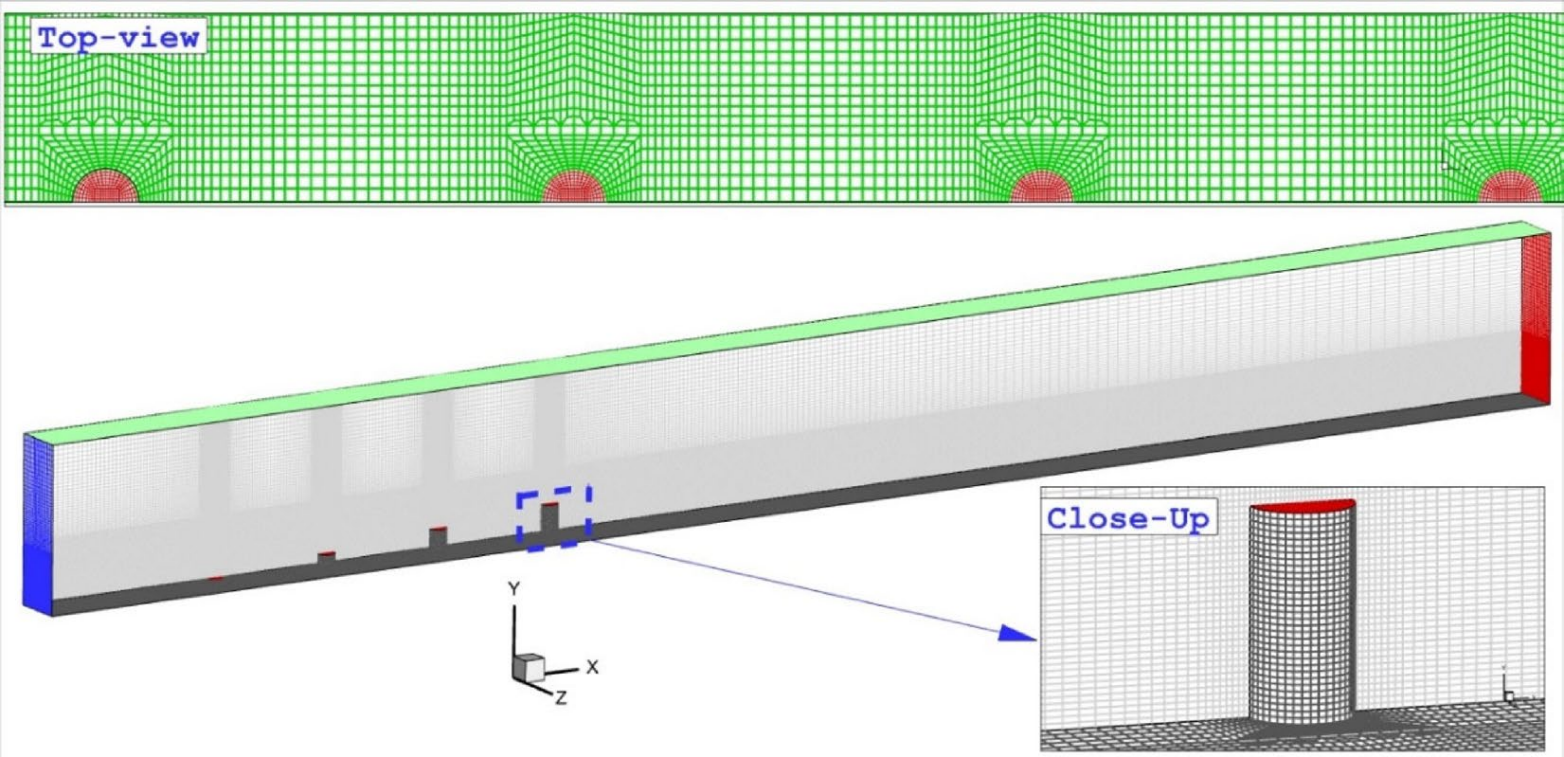
Applied grid for the chosen model.

The boundary conditions for our suggested configurations are displayed in Fig. 1. The pressure far-field is applied for the compressible air flow with Mach = 4 and static temperature of 1000 K at atmospheric pressure^43,44^. A wall with constant temperature is considered on the bottom and top of the domain. The hydrogen is our fuel type and it is injected through four injectors at different heights of 0, 0.25 mm, 0.5 mm and 0.75 mm from the bottom of the domain. The fuel jets with a total pressure of 10% of the main free stream is applied. As displayed in Fig. 2, three jet gaps of 4Dj, 7Dj and 10Dj are investigated since jet spaces have great impacts on the role of the circulation in the multi-jet configurations. The dimension of the domain along the x, z and y is 100 mm, 5 mm and 8 mm, respectively.

**Figure 3:**
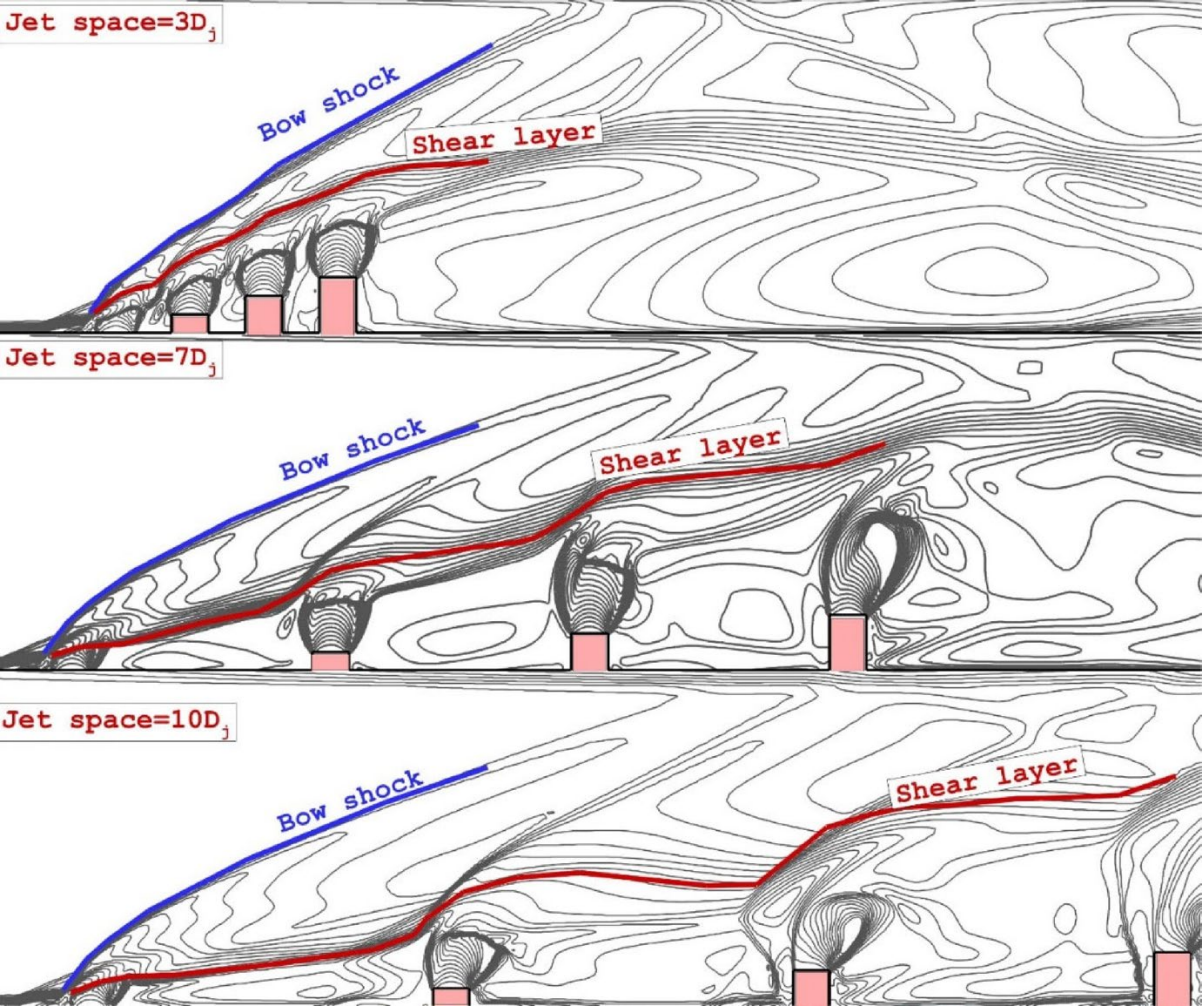
Mach contour on a jet plane.

The produced grid is also demonstrated in Fig. 2. As the fuel jet injection in cross-flow produced shock interactions, the resolution of produced grid near the tip of the jet nozzle is increased for shock capturing. The top view of the grid in Fig. 3 shows that the structured grids are generated in this study. It is also found that the applied grid is uniform in the whole domain to avoid errors due to grid non-uniformity. A grid study is also done in this investigation for the grid independency examination as presented in Table 1. In this table, the fuel mass concentration for the four produced grids on the definite surface positioned downstream of the injectors are compared. The presented data indicate that the fine grid is satisfactory for the coming simulations.

**Table 1:** Grid study.

	Cells	Cell num. along X, Y and Z direction	Hydrogen fraction at 35 mm downstream
Coarse	933,000	175 × 115 × 50	0.2630
Medium	1,620,000	195 × 145 × 60	0.2821
Fine	2,224,000	215 × 175 × 70	0.2881
Very fine	3,810,000	240 × 205 × 80	0.2891

## Results and discussion

### Authentication

Assessment of the numerical data with practical work is also done for the valuation of the correctness of attained results^45–47^. In this work, the value of the penetration height downstream of a single injector with a diameter of 2 mm is equated with further scientific works. Our data is equated with other works. The trend of the results and deviation from other investigations indicate that the obtained results are rational^48,49^.

### Flow analysis

The interface between jets is crucial for fuel mixing in a multi-jet system. Figure 3 demonstrates the impact of three distinct jet gaps on the interaction of fuel jets within the combustion chamber, where fuel injectors with similar heights are extruded. The first important factor related to these configurations is the angle of the bow shack. It seems that the bow shock angle of the model with jet space = 3Dj is higher than other arrangements. The variation of the shear layer is also highlighted in the figure to trail the jet feature is the symmetry plane where the main interactions happen. It is observed how jet spaces and upstream jet influence the deflection of barrel shocks. Increasing the jet spaces strengthen the deflection of the barrel shocks.

**Figure 4:**
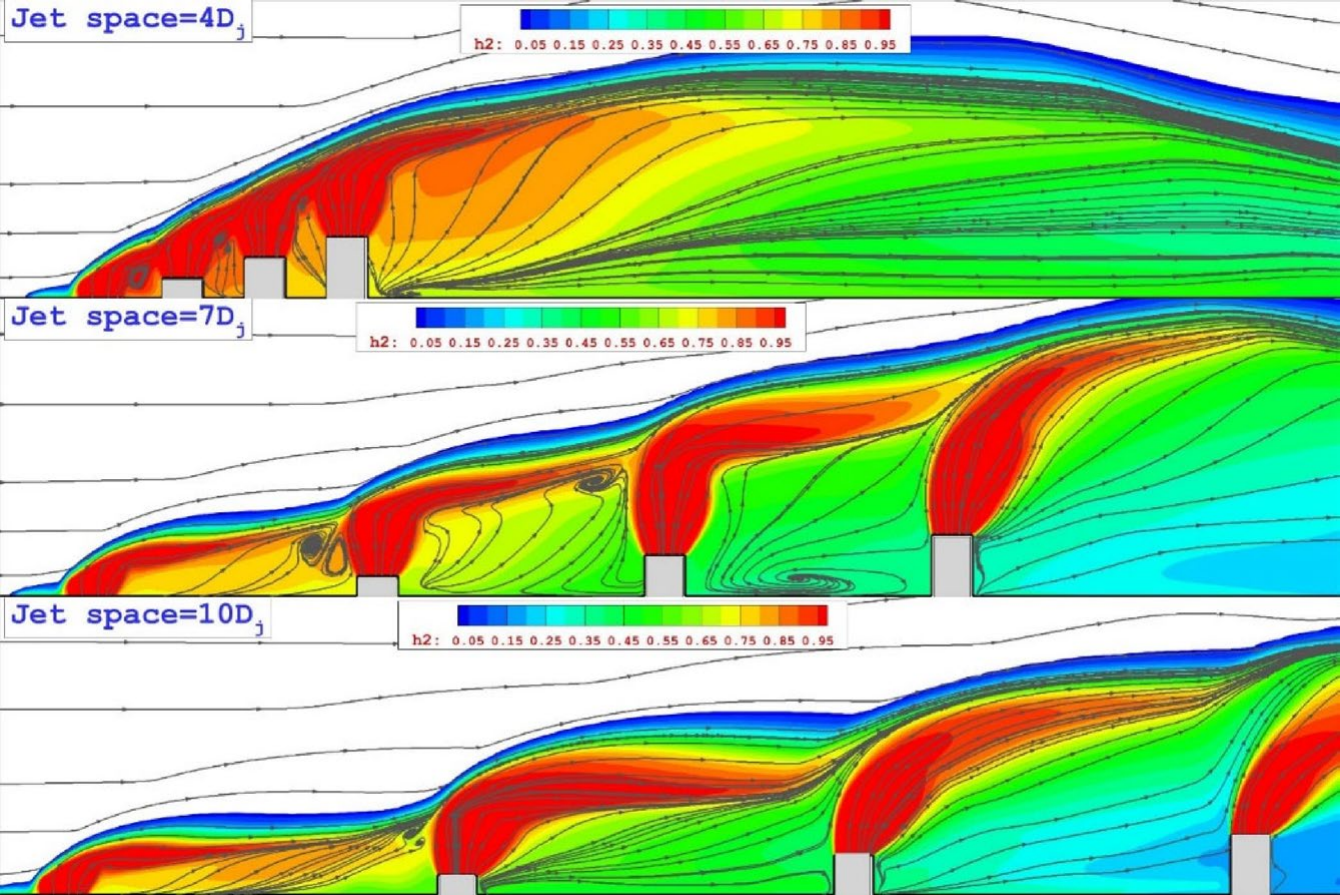
Evaluation of the mixing area on the jet plane.

The stream and mass concentration of these configurations are displayed in Fig. 4. The concentration of the hydrogen jets is high near the configuration with less jet space while jet configurations with high gap spaces result in less fuel concentration in the gap of extruded injectors. The figure also displays that the vortex structure has more pronounced effects in high jet gaps. In the jet space of 7Dj, the altitude of the mixing area is more uniform than other configurations while fuel distribution is done at a wider distance.

**Figure 5:**
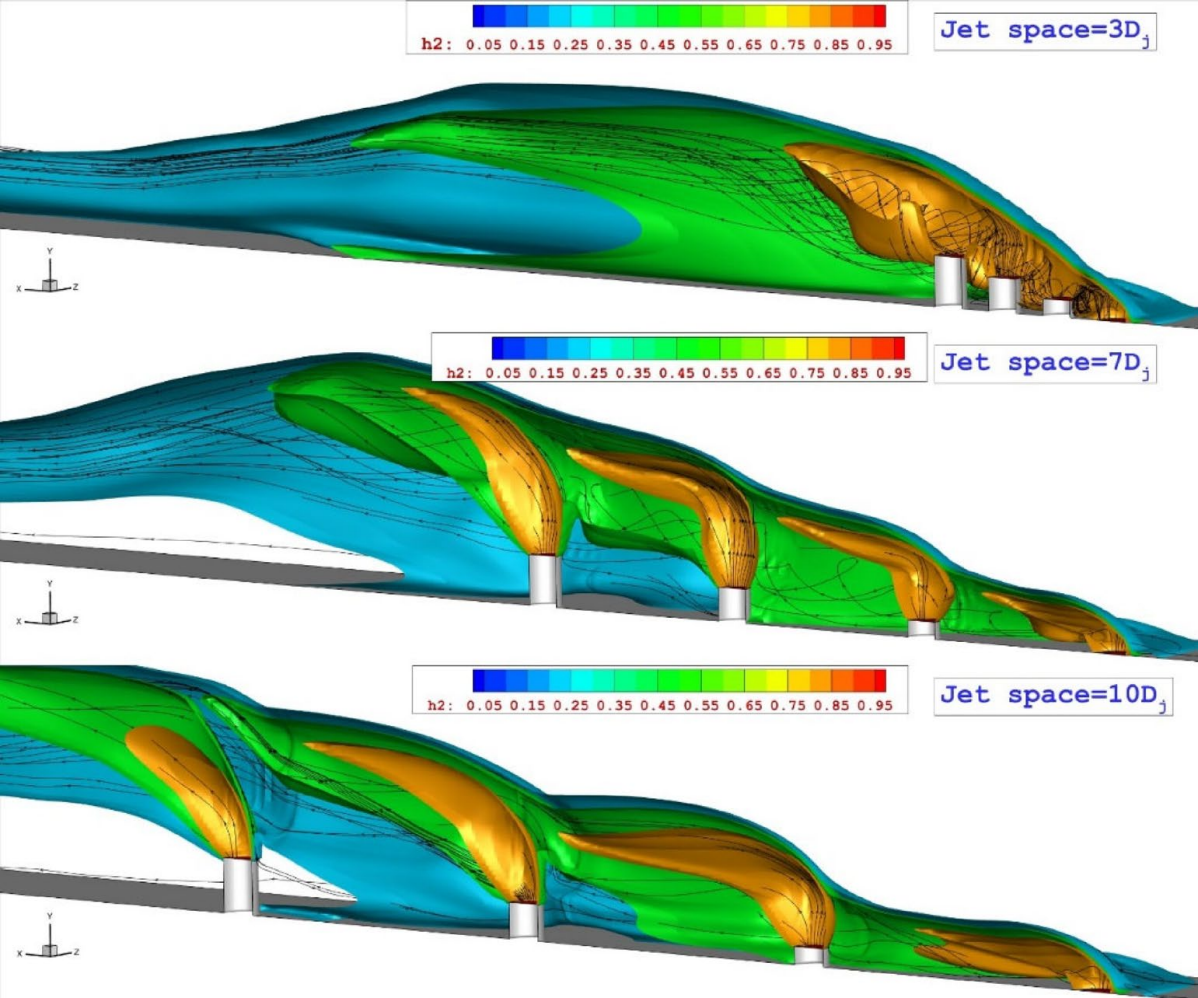
association of the fuel jet interactions for unlike jet spaces.

**Figure 6:**
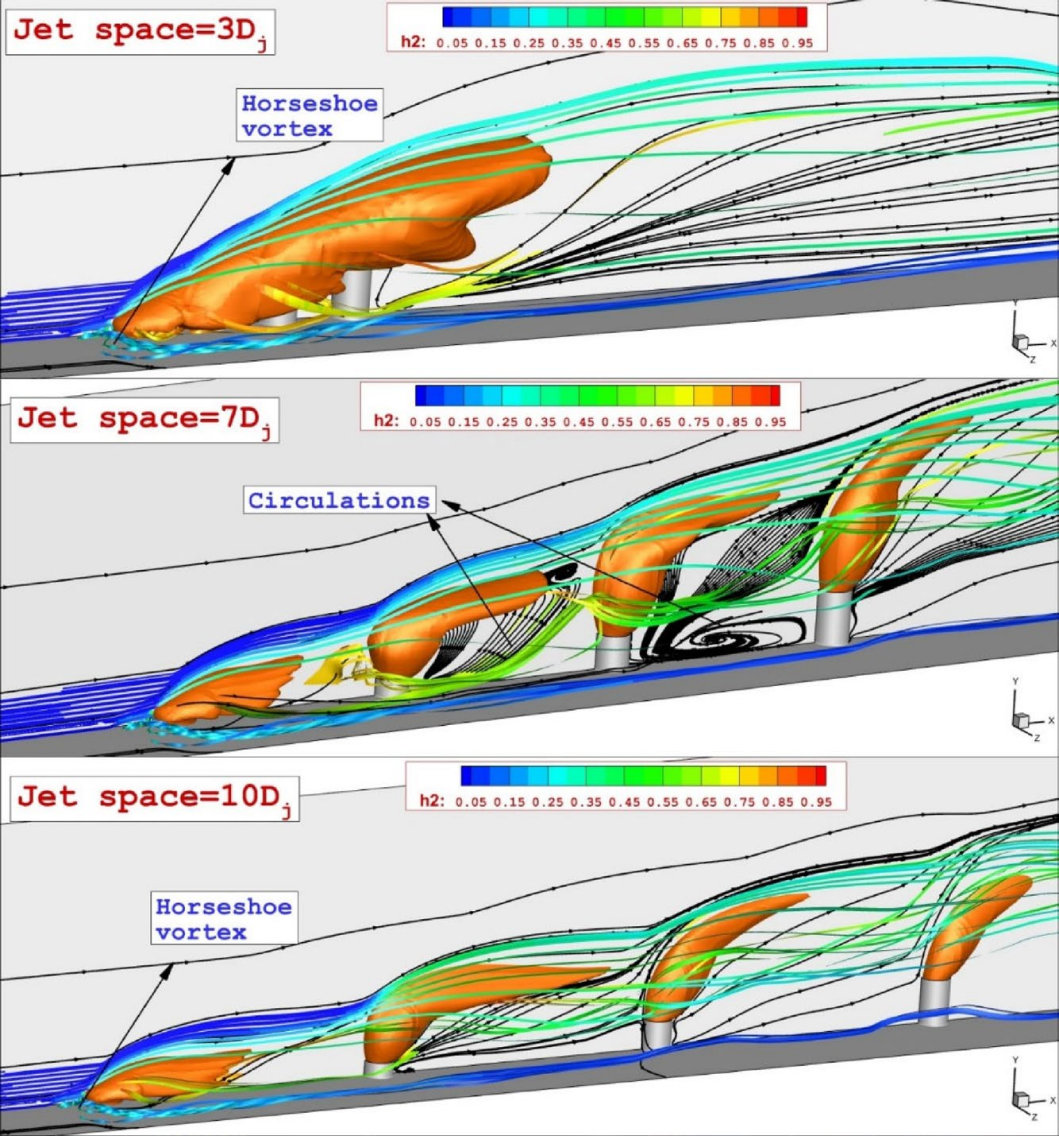
Evaluation of the flow stream and its impact on the fuel mixing mechanism.

The 3-D visualization of the jet stream in the selected configurations are illustrated in Fig. 5. The feature of the jet and stream indicates that the flow vortex is more visible in the model with small jet spaces. The jet interactions are higher in the low jet spaces as the barrel shock is closer. Figure 6 illustrates the flow hydrodynamics with a focus on circulations within the jet gap. This structure is dominant in the configuration with a jet gap of 7Dj. Increasing the jet space does not confine the flow within the extruded nozzles. In addition, a lower gap limited the mass flow rate within the extruded nozzles and this restricted its impacts on the jet stream and spreading in the combustion chamber. Multi-jet configurations are known for producing a horseshoe vortex upstream of the first nozzle. The size and strength of this vortex are influenced by the interface between the first jet and the mainstream. As previously mentioned, when the lower jet spacing is reduced, the jets combine to act as an extended jet, enhancing the interaction with the free stream and causing the horseshoe vortex to extend further into the domain. This results in improved fuel mixing near the injectors.

**Figure 7:**
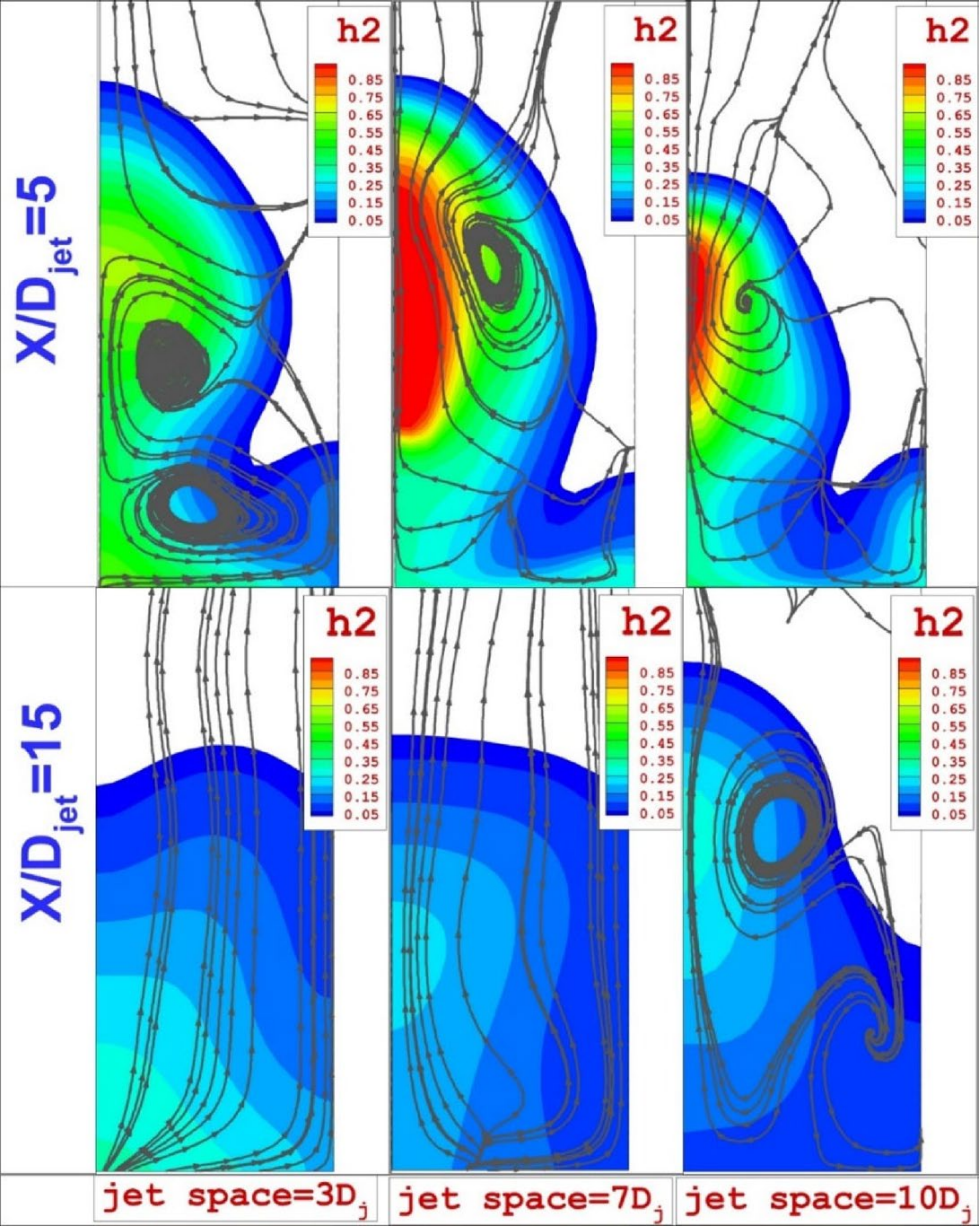
Estimation and comparison of the mixing zone and flow on planes downstream of the extruded nozzles.

Figure 7 displays the mixing zone and flow structure near the injector (x/Djet = 5) and far distance (x/Djet = 15). In the vicinity of injectors, there are two distinctive vortices that are more noticeable in the low jet space of 3Dj. The upper and the lower vortex are made by the communication of the jets with the mainstream. As described in the previous paragraph, the lower one is owing to the horseshoe vortex and its strength is related to the connections of the supersonic air stream with first jets. The upper vortex is related to the velocity difference due to the production of the vortex in the gap of extruded nozzles. In far distances, the impacts of the jet arrangements are not noticeable (Fig. 7).

### Quantitative evaluation

**Figure 8:**
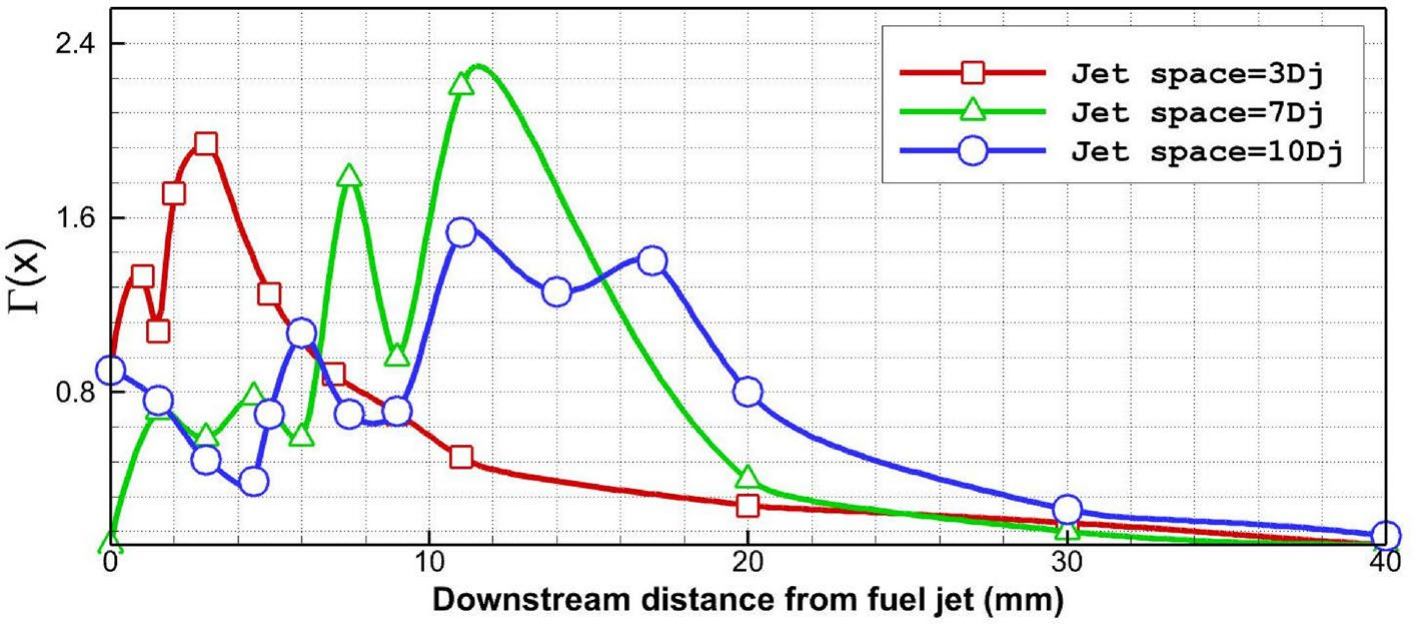
Difference of the circulation factor along the jets.

Figure 8 compares the change of circulation factor near the injectors as well as downstream extruder nozzles. Achieved results indicate that the maximum circulation strength was noticed in the model with a jet gap of 7 Dj. In fact, decreasing the jet space to less than 7Dj limited vortex effects while increasing the gap of injectors would not allow the formation of the vortex in jet gaps as noticed in the trend of circulation factor in the gap of the last two jets. Besides, the circulation strength is diminished in far distances for all cases.

**Figure 9:**
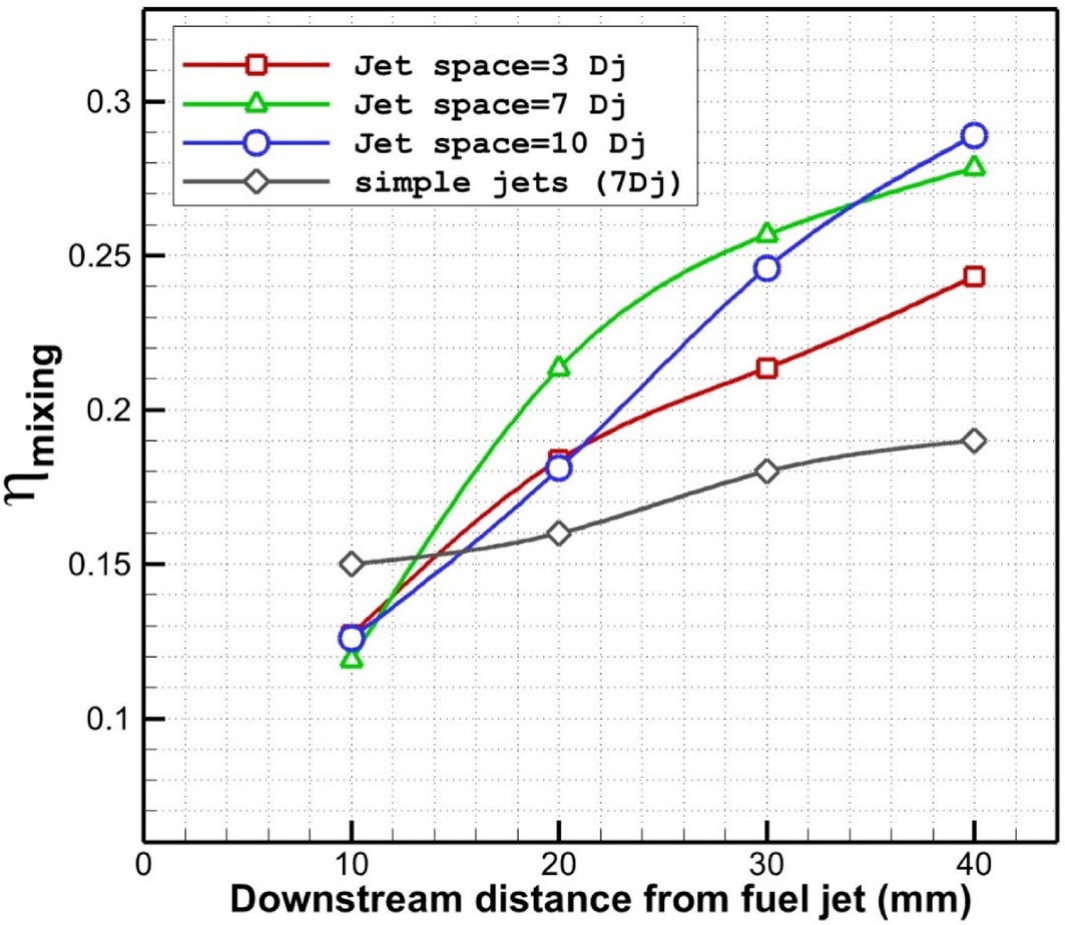
Role of jet spaces on the mixing performance of extruded nozzles.

Figure 9 shows the disparity of the fuel mixing downstream of the different gaps of extruded nozzles along with simple multi-jet configuration without extruded nozzles. Increasing the jet gap would increase the mixing in the far distance while all jet has similar mixing efficiency near the first nozzle. Comparison of extruded nozzle configuration with a simple nozzle show that the application of the extruded nozzle and the same jet space would increase the fuel mixing about 42% in the distance of 25 mm to 40 mm behind the first nozzle. This shows the influences of vortex formation within the gap of the injector. The normal mixing performance of the model with jet space of 7 Dj and 10 Dj in distances of 30 mm to 40 mm is almost equal. However, mixing enactment of the case with a gap of 7Dj is about 12% more than 10 Dj.

## Conclusion

The impacts of the extruded nozzle on fuel mixing of multi-circular jets are fully investigated at supersonic cross flow. The impacts of jet spaces on the size of the mixing area are revealed and the mechanism of the fuel distribution by the extruded nozzle are presented. CFD is applied for the visualization of the high compressible flow with cross multi-jets. The influences of jet spaces on the strength of the mixing and circulation efficiency are examined and the mixing effects of multi-extruded nozzles with simple ones are compared with full details. A comparison of the extruded nozzles with simple ones confirms the higher mixing efficiency of extruded nozzles. According to our findings, the lower jet spacing restricts the formation of vortices within the gap. Conversely, increasing the nozzle gap reduces the strength of the vortex, even when using extruded nozzles. We have observed that the mixing mechanism is closely tied to the presence of strong vortices at the jet distance.

## Data Availability

The datasets used and/or analyzed during the current study are available from the corresponding author on reasonable request.
